# Sleep Disturbances Associated With Hidden Hearing Loss: Insights From Human Data and a Mouse Model of Sleep Fragmentation

**DOI:** 10.1002/brb3.70778

**Published:** 2025-08-27

**Authors:** Xiaodi Wang, Bo Su, Jiaxin Zhang, Yingqiang Li, Liangqiang Zhou, Zhihui Du, Hanqi Chu, Huifang Liang, Dan Bing

**Affiliations:** ^1^ Department of Otolaryngology‐Head and Neck Surgery, Tongji Hospital, Tongji Medical College Huazhong University of Science and Technology Wuhan P. R. China; ^2^ Hepatic Surgery Centre, Tongji Hospital, Tongji Medical College Huazhong University of Science and Technology Wuhan Hubei P. R. China

**Keywords:** auditory nerve, hidden hearing loss, inner hair cell, ribbon synapse, sleep fragmentation

## Abstract

**Background:**

Sleep disorders have been associated with auditory dysfunction. However, the specific effects of sleep fragmentation (SF) on the peripheral auditory system and the underlying mechanisms remain unclear.

**Methods:**

Data from the National Health and Nutrition Examination Survey (NHANES) were analyzed to examine the association between sleep disturbances and difficulties in speech perception in noisy environments among participants with normal audiograms. Additionally, an SF mouse model was used to assess the impact of sleep disruption on auditory function and cochlear pathology. Auditory brainstem response (ABR) testing, immunofluorescence staining, and transmission electron microscopy were performed to evaluate the auditory system.

**Results:**

NHANES data revealed that individuals with sleep disorders, even with normal audiograms, were more likely to experience difficulties in speech perception in noisy environments. In the mouse model, chronic SF led to a significant decrease in the amplitude and a prolonged latency of ABR wave I, without detectable changes in hearing thresholds. Immunofluorescence staining revealed a significant decrease in the number of ribbon synapses in inner hair cells and an increase in orphan ribbons. Moreover, electron microscopy demonstrated myelin damage in the auditory nerve fibers.

**Conclusions:**

Sleep fragmentation induces subtle damage to the auditory system, particularly affecting inner hair cell synapses and auditory nerve fibers, which may underlie difficulties in speech recognition in noisy environments, a potential indicator of hidden hearing loss.

## Graphical abstract

By leveraging both human and animal data, it uncovers the impact of poor sleep on hearing in humans and critical pathological changes in the sleep‐fragmentation mouse cochlea. This offers new directions for future research and potential interventions in sleep and auditory health.

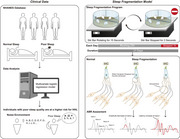

## 1 Introduction

Modern society places increased pressure on individuals, intensifying the pace of life and impacting their well‐being (the Stress in America 2020 survey signals a growing national mental health crisis 2020). Consequently, many people grapple with sleep disorders such as insomnia and fragmented sleep, resulting in a significant decline in sleep quality (Conte et al. 2022; Nelson et al. 2022). Poor sleep has been linked to an increased risk of various ailments, including liver cancer, cardiovascular diseases, and Alzheimer's disease (Yang et al. 2023; Irwin and Vitiello 2019; Miller and Howarth 2023). Moreover, these conditions are believed to be connected with the onset of auditory disorders. Multiple clinical studies have highlighted the detrimental effects of sleep disorders on the auditory system. For instance, an observational study suggests that SSNHL is associated with an increased incidence of insomnia (Yeo et al. 2022). Furthermore, extensive surveys have revealed a correlation between sleep disorders and an increased incidence of hearing loss on a larger scale (Lee et al. 2023; Wang et al. 2025).

In clinical settings, it is noted that not all individuals presenting with sleep disorders exhibit measurable alterations in auditory thresholds, despite self‐reported disruptions in auditory perception, particularly in the context of noisy environments. Traditionally, such auditory perceptual deficits have been attributed to pathological alterations within the central auditory pathway (Brisson and Tremblay 2021; Price and Bidelman 2021). Nevertheless, the conceptualization of hidden hearing loss (HHL) by Kujawa and Liberman in 2009 (Kujawa and Liberman 2009) offered an alternative etiology for diminished speech recognition in noise, one that is not centrally mediated. Their work proposed that exposure to excessive noise or the aging process could precipitate HHL through the impairment of inner hair cell (IHC) ribbon synapses (Kujawa and Liberman 2009; Monaghan et al. 2020; Wu et al. 2019), thereby providing a new perspective on the impact of peripheral auditory system pathology on auditory function in complex listening environments. Previous research suggests that long‐term sleep disturbances frequently result in multiple noticeable injuries to the central nervous system, thereby facilitating the progression of neurodegenerative disorders like Alzheimer's disease (Parhizkar et al. 2023). However, it remains uncertain whether speech recognition impairments related to sleep disorders can be partially attributed to the aforementioned pathological changes in the peripheral auditory system.

Given the difficulty in obtaining human temporal bone specimens, and following the preliminary observation of an association between sleep disturbances and impaired speech recognition in noisy conditions among individuals with normal audiograms, as identified through the National Health and Nutrition Examination Survey (NHANES) database, we developed a mouse model of sleep fragmentation. This model was employed to investigate the presence of pathological changes in the ribbon synapses of IHC and auditory nerve fibers (ANF) in subjects without measurable hearing loss. The objective of this study was to elucidate potential mechanisms underlying the diminished capacity for speech recognition in noisy environments among individuals suffering from sleep disturbances.

## 2 Materials and Methods

### 2.1 Ethics Statement

Animal experiments were conducted according to the protocol approved by the Laboratory Animal Welfare & Ethics Committee of TongJi Hospital (Wuhan, China) (Approval no. TJH‐202204023). Ethical approval for the NHANES protocol was secured from the National Center for Health Statistics ethical review board for human subjects. All participants provided written informed consent and received compensation, as well as a comprehensive medical report. Our study adhered to the Strengthening the Reporting of Observational Studies in Epidemiology (STROBE) reporting guidelines.

### 2.2 Human Study

#### 2.2.1 Study Design and Participants

In our study, we utilized data from the 2011–2012 and 2015–2016 cycles of NHANES. The NHANES, an initiative of the National Center for Health Statistics, is designed to evaluate the health and nutritional status of the U.S. populace. Utilizing data from the U.S. Census, the program selects a sample comprised of individuals who are not residing in institutional settings within the United States (Lou 2025). Our analysis focused on a subset of 18,883 participants who underwent both interviews and assessments at Mobile Examination Centers (MECs). The inclusion criteria were restricted to individuals aged between 20 and 69 who had completed a pure‐tone audiogram and had answered specific questions relating to audiometry and sleep disorders. To define speech‐in‐noise (SiN) perception impairment in a population with normal audiograms, we combined results from pure‐tone audiograms with self‐reported issues related to SiN perception. According to World Health Organization guidelines, a PTA of 20 dB or higher in the better hearing ear was categorized as abnormal. We excluded individuals with abnormal audiograms from our sample, resulting in a final sample size of 5433 participants who had complete data on audiology, sleep, and other variables of interest (Supplementary Figure S1).

#### 2.2.2 Hearing Assessment

Audiometric testing was conducted in noise‐controlled booths within MECs using an AD226 audiometer and TDH‐39 over‐ear headphones to measure hearing levels across seven frequencies from 0.5 to 8 kHz. Alternative equipment like E•A•Rtone 3A insert earphones was used for participants with asymmetrical hearing loss or problematic ear canals. Individuals unable to remove hearing aids or experiencing significant discomfort were excluded. The average hearing threshold, known as the pure‐tone average (PTA), was computed across frequencies of 0.5, 1, 2, and 4 kHz. The study also employed a self‐report questionnaire to assess SiN perception by asking participants, “How often do you find it difficult to follow a conversation if there is background noise, for example, when other people are talking, the TV or radio is on, or children are playing? Would you say…?” Response options were “Never,” “Seldom,” “About half the time,” “Usually,” or “Always,” and those who selected “About half the time,” “Usually,” or “Always” were classified as having SiN perception issues (Supplementary Table S1).

#### 2.2.3 Sleep Assessment

Sleep trouble was assessed by asking participants if they had ever told a doctor or health professional that they have trouble sleeping. Sleep disturbance was determined based on responses to the question, “Over the last 2 weeks, how often have you been bothered by trouble falling or staying asleep, or sleeping too much?” Participants who answered “more than half the days” or “nearly every day” were considered to have sleep disturbance, while those who answered “several days” or “not at all” were considered to have no sleep troubles. (Yin et al. 2023).

#### 2.2.4 Covariates

The study considered several demographic and health‐related variables. Age was categorized into two groups: under 60 years and 60 years or older, and participants were classified by gender as male or female. Race/ethnicity was delineated as Hispanic, non‐Hispanic Black, non‐Hispanic White, and other. Educational background was segmented into three categories: less than high school, high school graduate, and education exceeding high school. Body Mass Index (BMI) was divided into three ranges: < 25, 25–30, and ≥ 30 kg/m^2^. Auditory‐related variables included tinnitus—defined as experiencing ringing, roaring, or buzzing in the ears or head lasting at least 5 min in the past 12 months—and noise exposure, determined by affirmative responses to ever having been exposed to very loud noise at work or off‐work. Medical history encompassed high blood pressure, defined as the use of antihypertensive medication or an average blood pressure of 140/90 mm Hg or higher (obtained from three separate measurements), a history of cardiovascular diseases (positive response to questions about congestive heart failure, coronary heart disease, angina/angina pectoris, heart attack, or stroke), and a history of respiratory diseases (positive response to having chronic bronchitis and/or emphysema).

### 2.3 Animal Study

#### 2.3.1 Sleep Fragmentation Mice Model

Male C57BL/6J mice, aged 6–8 weeks, were acquired from the Experimental Animal Center at Tongji Hospital, Wuhan. Before initiating sleep fragmentation, the mice were acclimatized to the device over three days. Based on our literature review, the estrous cycle in female mice can introduce significant variability in circadian rhythms (Nolan 2020) and potentially modulate the effects of sleep fragmentation on auditory function. Additionally, estrogen has been reported to exert protective effects on hearing (Delhez et al. 2020). To minimize within‐group variability and confounding effects of hormonal fluctuations, we opted to use male mice, which exhibit more stable physiological responses, as our experimental animals. This was followed by a three‐week period of SF, during which the device was operational for 23 h daily, with a 1‐h pause. The bar was programmed to move at speed 5 and to alternate between clockwise and counterclockwise rotations. The control group mice were housed in SF apparatuses without rotating rods, with all other conditions identical to those of the SF group mice. For detailed video footage of the SF mouse model, please refer to the supplementary materials.

#### 2.3.2 Auditory Brainstem Response Assessment

Mice were anesthetized via an intraperitoneal injection containing 1.25% tribromoethanol (20 µL/g) and situated on a temperature‐controlled pad. Electrodes were strategically positioned as follows: the reference electrode beneath the left ear, the recording electrode at the center of the cranium, and the ground electrode above the mouse's right thigh. These electrodes were subsequently connected to an auditory evaluation instrument by means of a data cable. The Auditory Brainstem Response (ABR) testing apparatus (Giant Tek, GAT‐ABR365) facilitated sound stimulation, evoked potential amplification, data acquisition, and processing functions. Click stimulus primarily assessed broad‐spectrum auditory capabilities, while tone burst stimulus targeted specific frequency perception, encompassing 4k, 8k, 12k, 16k, 24k, and 32 kHz, decrementing from 90 to 10 dB in 10 dB intervals. Each intensity level was repeated 500 times, with the accumulated waveforms forming the auditory signal. As sound intensities diminished, auditory wave amplitudes decreased until waveforms were indiscernible. This specific sound intensity was deemed the threshold for the given frequency. All auditory examinations were performed in an acoustically isolated environment. The data readers were blinded to the animal group assignments.

#### 2.3.3 Cochlea Immunofluorescence Staining and Image Analysis

Following the ABR assessment, all mice were anesthetized and sacrificed. Cochleae were fixed in 4% paraformaldehyde at ambient temperature for 1 h and subsequently decalcified using 10% EDTA until the semicircular canals became unbreakable using forceps. Parts of cochleae were dissected to isolate the basilar membrane, which was adhered to a circular coverslip utilizing Cell‐Tak (Corning, 354240) for whole‐mount immunofluorescence. The segmentation and observation sites of the basilar membrane were shown in Supplementary Figure S2. Other parts of the cochleae underwent dehydration in a 30% sucrose solution at 4°C overnight, positioned in optimal cutting temperature (Sakura, 4583) compound at 4°C overnight. While sectioning, the cochlea was placed in the sample holder's center and promptly frozen using a freezing microtome (Leica CM1900), followed by 6 µm sectioning.

For immunofluorescence staining, tissues underwent permeabilization with 0.5% Triton‐X solution for 30 min and blocked with 10% goat serum for 10 min. Subsequently, they were incubated with primary antibodies overnight at 37°C. Primary antibodies included chicken anti‐neurofilament H (1:200, Millipore, AB5539) for neurofibers, rabbit anti‐myelin basic protein (1:100, Abcam, ab218011) for myelin sheaths, mouse IgG1 anti‐CtBP2 (1:200, BD, 612044) for ribbon, and mouse IgG2a anti‐GluR2 (1:2000, Millipore, MAB397) for glutamate receptor. After washing three times with phosphate‐buffered saline (PBS), tissues were incubated with secondary antibodies in darkness for 1 h. For ribbons observation, the tissue and secondary antibody were incubated at 37°C twice, each time for 1 h. Secondary antibodies utilized were goat anti‐chicken 488 (1:100, Abcam, AB150169), goat anti‐rabbit Cy3 (1:100, Abclonal, AS007), Alexa Fluor‐488‐coupled goat anti‐mouse IgG1 (1:1000, Thermo, A‐21121), and Alexa Fluor‐568‐coupled goat anti‐mouse IgG2a (1:1000, Thermo, A‐21134). For outer hair cell observation, tissues were additionally incubated with Alexa Fluor‐488‐coupled phalloidin (1:100, Servicebio, G1248‐100T). Tissues were then sequentially washed three times with PBS, stained with DAPI (Abclonal, RM02978) for nuclei, and sealed using anti‐fade mounting medium (Servicebio, G1401‐5ML). Employing a laser confocal microscope (Olympus, FV1000), multilayer Z‐axis confocal images were captured. These images were subsequently amalgamated and projected utilizing ImageJ (NIH software) to analyze outer hair cells and count ribbon synapses within inner hair cells.

For quantifying the ribbon synapses of inner hair cells, three levels of frozen sections were selected from each cochlea. The ribbon synapses of inner hair cells in the apical, middle, and basal turns were counted, and the average value was taken as the number of ribbon synapses for that mouse. Data from three mice per group were used for statistical analysis.

#### 2.3.4 Preparation and Imaging of Transmission Electron Microscopy Specimens

After the mice were anesthetized, they were euthanized, and the cochlea was extracted. Any excess tissue was removed in electron microscope fixative. A hole was drilled at the apex of the cochlea, which was then perfused with the fixative. The cochlea was stored at 4°C overnight. The sample was decalcified using a 10% EDTA solution for two days, with the solution being changed daily. The basilar membrane was dissected out, and the tectorial membrane was removed. The basilar membrane was dehydrated using a graded alcohol series and then infiltrated with a 1:1 mixture of acetone and EPON 812 for 40 min. It was subsequently infiltrated with an embedding agent and DMP‐30 for 12 h. The basilar membrane was embedded in epoxy resin using molds. The molds were polymerized in an oven at 35°C for 12 h, 45°C for 12 h, and 60°C for 24 h. Semi‐thin sectioning was performed for positioning, followed by ultra‐thin sectioning. The sections were stained with saturated uranyl acetate solution for 30 min and lead citrate for 15 min. The specimens were observed under a transmission electron microscope (HITACHI, HT7800) at magnifications of 2500× and 5000×.

#### 2.3.5 Analysis of Myelin Sheath Thickness in Auditory Nerve Fibers

The thickness of the myelin sheath in the auditory nerve fibers of mice was quantified using the method described in a previous study (Wan and Corfas 2017). In brief, the longest transverse diameter perpendicular to the long axis of the cross‐section of the auditory nerve was taken as the diameter d1 of the nerve fiber. The diameter d2 of the axon, excluding the myelin sheath, was measured along this diameter d1. The g‐ratio was calculated as d2/d1. A higher g‐ratio indicates a thinner myelin sheath in the auditory nerve. Three fields of view were taken from each cochlea to calculate the g‐ratio of the nerve fibers, and the average g‐ratio from these fields was used as the g‐ratio value for the corresponding mouse. The g‐ratio values of three mice from each group were used for statistical analysis.

### 2.4 Data Analyses

In our human study, all analyses considered the weighted design of NHANES. Categorical variables were described using raw counts and percentages, and Rao–Scott chi‐square tests were employed for group comparisons. Multivariate logistic regression was used for the binary outcome, adjusting for age, sex, race/ethnicity, BMI, comorbid conditions (cardiovascular diseases, hypertension, respiratory diseases, and diabetes), and hearing‐related factors (noise exposure and tinnitus). Missing data were handled through a complete case approach. Results from regression models were reported as odds ratios (ORs), supplemented by 95% confidence intervals (CIs) and *p*‐values. Subgroup analyses were performed to assess variations in the relationships across subgroups and conditions. Interaction terms were incorporated into the models to evaluate the significance of effect modification. Data analysis utilized the ‘survey’ package in R (http://www.R‐project.org), executed in RStudio (Version: 2023.09.1+494, RStudio, Inc., Boston, MA). A two‐sided p‐value below 0.05 was considered statistically significant.

For the animal study, the experiment data were presented as means ± standard deviation. The unpaired *t*‐test in GraphPad Prism software (GraphPad Prism version 9.5.1 for Windows, GraphPad Software, Boston, Massachusetts, USA, www.graphpad.com) was employed to compare data across different groups, considering a p‐value of less than 0.05 as statistically significant.

## 3 Results

### 3.1 Patients With Sleep Disorders and Normal Audiograms May Demonstrate a Greater Difficulty in Speech Recognition in Noise

Out of 5433 individuals studied, 1273 (23.6%) exhibited a SiN perception impairment with normal audiograms. Participants were aged between 20 and 69 years, with an average age of 40.6 ± 0.4 years. The group consisted of 2886 women (53.4%). The data in Supplementary Table S1 shows that, when taking population weight into account, those with SiN perception problems were predominantly white (*p* = 0.001), had lower education levels (*p* = 0.001), had a hearing‐related history of noise (*p* < 0.001) and tinnitus (*p* < 0.001), and had sleep disturbance (*p* < 0.001) and sleep trouble (*p* < 0.001).

Table 1 illustrates the association between the perception of SiN perception problems and sleep conditions, analyzed using multivariate logistic regression across three models. Sleep disturbance was found to be significantly associated with SiN perception issues, with an OR of 1.883 (95% CI, 1.468–2.417) after adjusting for potential confounders, including age, sex, race/ethnicity, BMI, concurrent conditions (such as cardiovascular diseases, hypertension, respiratory diseases, and diabetes), and hearing‐related status (noise exposure and tinnitus). Additionally, sleep troubles were similarly related to SiN perception problems, yielding an OR of 1.907 (95% CI, 1.527–2.380) after adjustment. Furthermore, subgroup analyses indicated that these associations were significant for both exposures at least at one level within the subgroups examined (Supplementary Table S2).

**TABLE 1:** Relationship between SiN perception problem and conditions of sleep analyzed by multivariate logistic regression.

	Model 1^a^		Model 2^b^		Model 3^c^	
	OR (95% CI)	*p* value	OR (95% CI)	*p* value	OR (95% CI)	*p* value
Sleep disturbance	2.207 (1.776–2.743)	< 0.001	1.990 (1.585–2.498)	< 0.001	1.883 (1.468–2.417)	< 0.001
Sleep trouble	2.137 (1.725–2.648)	< 0.001	2.046 (1.634–2.562)	< 0.001	1.907 (1.527–2.380)	< 0.001

**Abbreviations**: CI, confidence interval; HHL, hidden hearing loss; OR, odds ratio.

^a^ Model 1 was adjusted for age, sex and race/ethnicity.

^b^ Model 2 was adjusted for age, sex, race/ethnicity and education.

^c^ Model 3 was adjusted for age, sex, race/ethnicity, BMI, concurrent conditions (cardiovascular diseases, HBP, respiratory diseases, diabetes) and hearing related‐status (noise exposure and tinnitus).

### 3.2 Chronic Sleep Fragmentation in Mice Leads to Reduced Amplitude and Prolonged Latency of ABR Wave I

**FIGURE 1:**
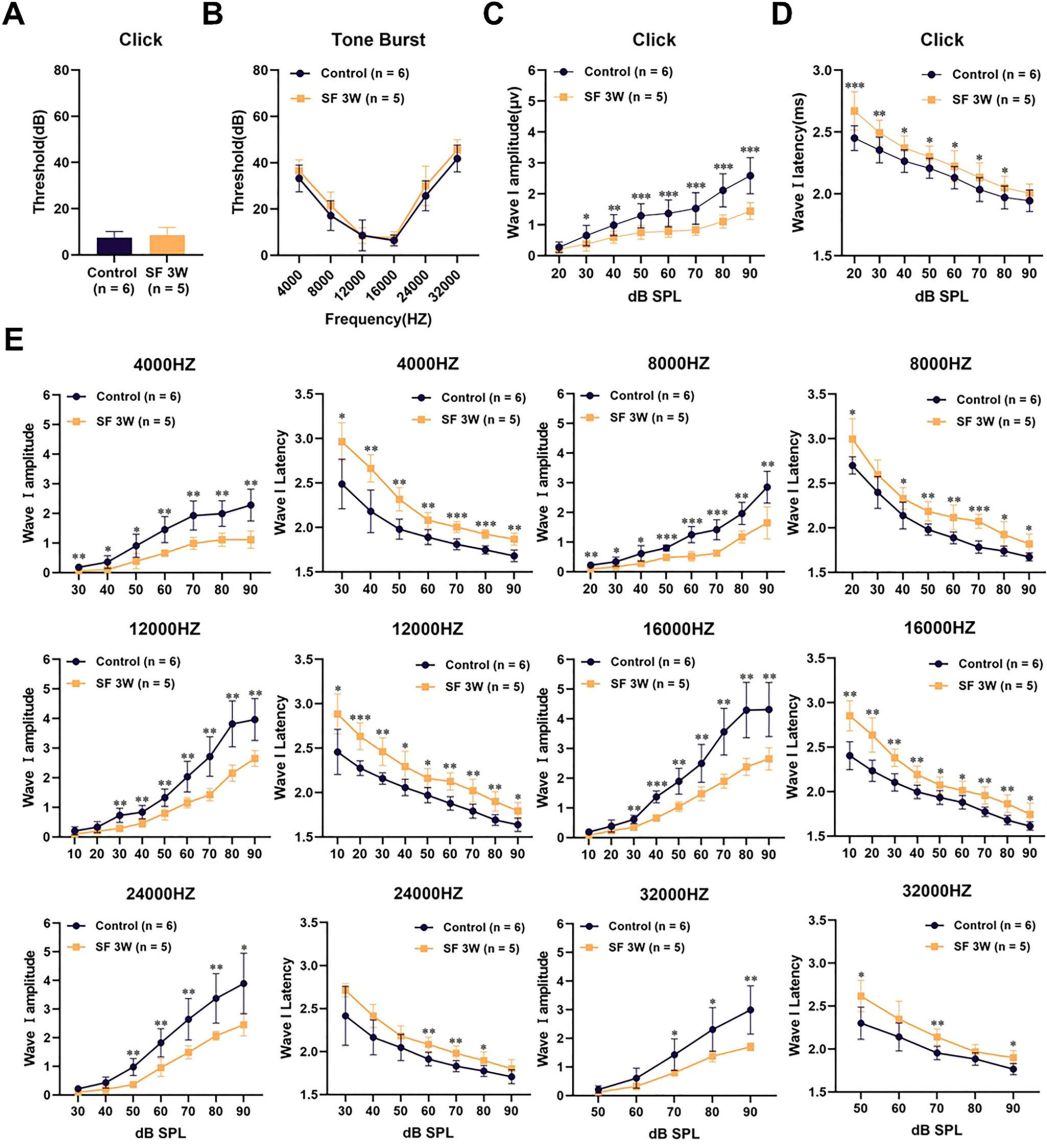
**Assessment of ABR following 3 weeks of sleep fragmentation. (A)** Click‐evoked ABR testing, **(B)** Tone Burst ABR testing at frequencies of 4k, 8k, 12k, 16k, 24k, and 32 kHz, **(C)** Measurement of ABR wave I amplitude, and **(D)** Analysis of ABR wave I latency. Group Comparison: Control (*n* = 6) vs. SF: sleep fragmentation (*n* = 5). *** *p* < 0.001, ** *p* < 0.01, and * *p* < 0.05.

To investigate the effects of SF on auditory function, ABR tests were administered to mice before and after a 3‐week SF period. Despite not showing explicit threshold shifts in ABR tests (Figure 1; A and B), these mice exhibited marked decreases in click ABR wave I amplitude (Figure 1C) and extended latency periods (Figure 1D). Next, we also analyzed ABR data under different frequency sound stimuli, which reflect different locations on the basilar membrane. The results indicated that, under all the frequency sound stimuli employed (4k8k、12k、16k、24k、32k), the amplitude of the ABR wave Ⅰ in mice subjected to 3 weeks of SF was significantly decreased, and the latency was significantly extended (Figure 1E). These observations indicate that mice with SF might exhibit subtle yet significant audiological changes in the whole cochlea, suggesting that SF leads to auditory dysfunction.

### 3.3 Chronic Sleep Fragmentation Leads to Damage in the Synapses of IHC and ANF

**FIGURE 2:**
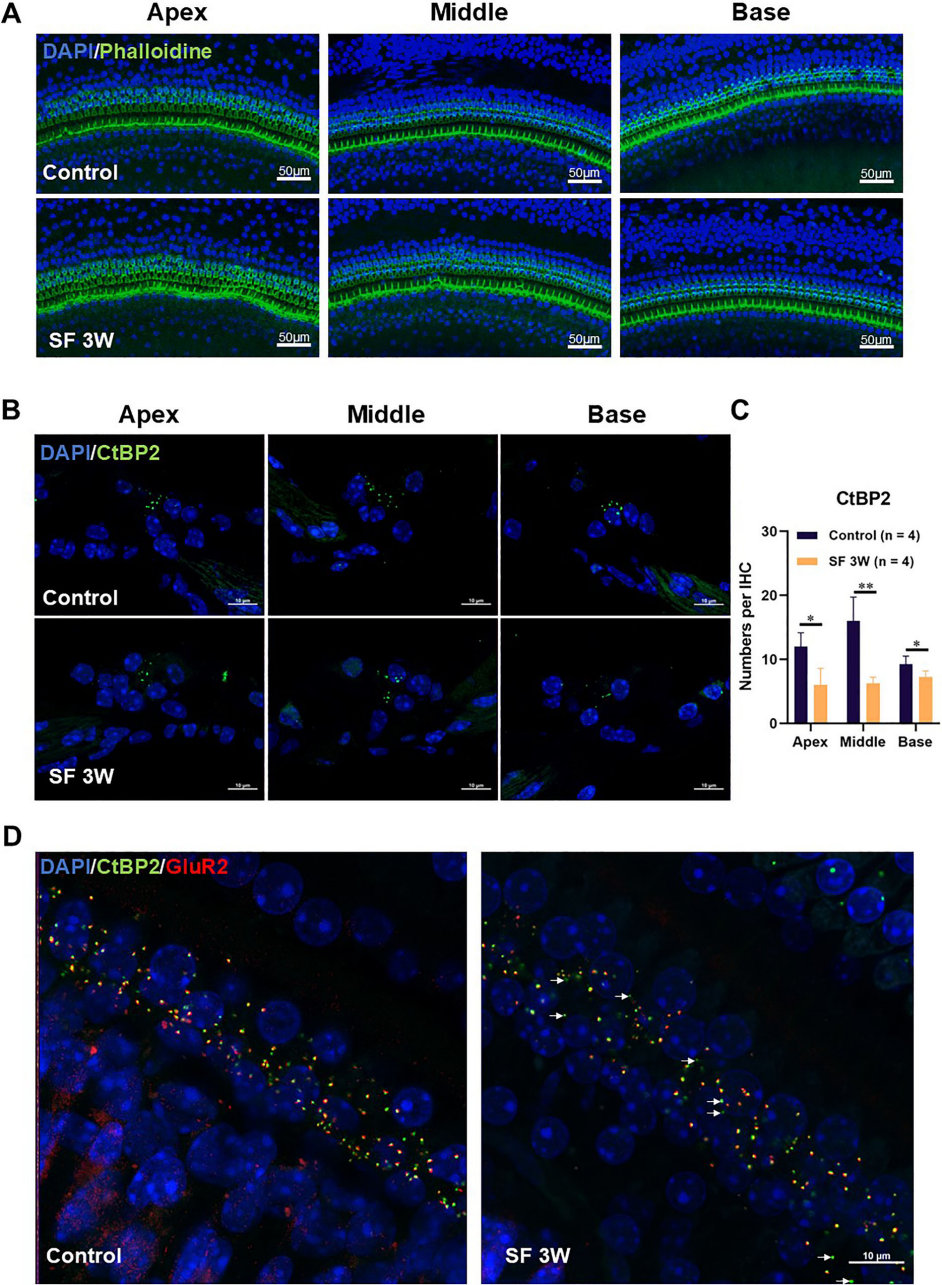
**Damage to mice IHCs after 3 weeks of sleep fragmentation. (A)** Observation of OHCs using whole‐mount basilar membrane preparation with DAPI (blue) nuclear staining and phalloidin (green) hair cell stereocilia bundles staining, **(B)** Immunofluorescent staining of frozen sections of ribbon synapses in IHCs. Ribbon marker CtBP2 (green), nucleus stained with DAPI (blue), **(C)** Counting of ribbon synapses in frozen sections of IHCs, and **(D)** The synapses of the basilar membrane were observed with the ribbon marker CtBP2 (green), post‐synaptic marker GluR2 (red), and nucleus marker DAPI (blue). The white arrows indicate the locations of orphan ribbons. Group Comparison: Control (*n* = 4) versus SF: sleep fragmentation (*n* = 4). ** *p* < 0.01 and * *p* < 0.05.

To investigate potential hair cell loss, we utilized a co‐staining approach with phalloidin, a marker for hair cell stereocilia bundles, and DAPI. Consistent with our auditory assessments, we observed no substantial loss of OHC in the cochlear whole‐mounts of SF mice without detectable hearing loss (Figure 2A). Furthermore, using CtBP2 immunofluorescence staining, we detected a reduction in the synapse count in SF mice compared to controls (Figures 2B; and C). To further evaluate the integrity of the synaptic connections, we performed immunostaining of the postsynaptic elements using antibodies targeting the AMPA‐type glutamate receptor GluR2. In a healthy ear, positive staining for GluR2 and CtBP2 typically forms perfect one‐to‐one pairings (Kujawa and Liberman 2015). The results revealed an increased proportion of orphan ribbons, which lacked apposed‐postsynaptic elements in the SF mice (Figure 2D; white arrows).

### 3.4 Chronic Fragmented Sleep Leads to the Damage to the Myelin Sheath of ANF

**FIGURE 3:**
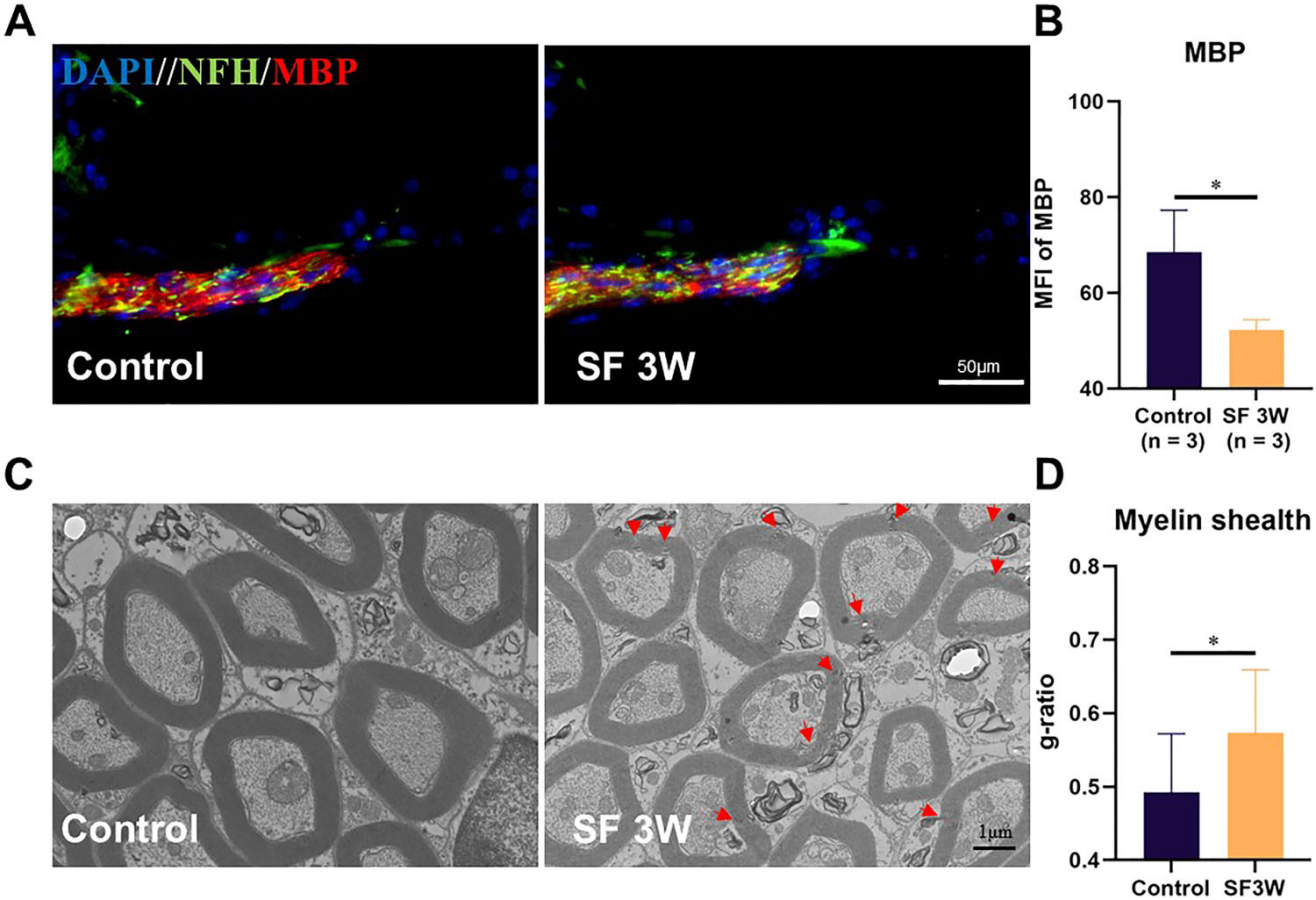
**The myelin sheath of mice ANF shows pathological changes and becomes thinner due to 3 weeks of sleep fragmentation. (A)** Detection of neural fiber myelin in cochlear frozen sections, nuclei stained with DAPI (blue), myelin marked with MBP (red), and neuro fibers marked with NFH (green), **(B)** Quantitative calculation of MBP immunofluorescence in Figure 3A. **Abbreviation**: MFI, mean fluorescence intensity; **(C)** Observation of cochlear ANF myelin sheath under a transmission electron microscope. The red arrow indicates the demyelinated area. d1 is the diameter of the auditory nerve fiber, and d2 is the diameter of the axon, and **(D)** Analysis of auditory nerve myelin sheath thickness. A larger g‐ratio (d2/d1) indicates a thinner myelin sheath. Group Comparison: Control (*n* = 3) versus SF: sleep fragmentation (*n* = 3). * *p* < 0.05.

In order to further investigate the impact of chronic SF on auditory nerve integrity in mice without detectable hearing loss, we conducted fluorescence staining of the ANF and their myelin sheath. Co‐staining with markers for nerve fibre (NFH) and their myelin basic protein (MBP) revealed a significant reduction in the fluorescence intensity of myelinated nerve fibers in SF mice, suggesting potential lesions in the myelin sheath of ANF (Figures 3A, B). To gain a more detailed understanding of the pathological changes in the myelin sheath, we examined their structure using transmission electron microscopy. Electron microscopy confirmed a significant number of myelin lesions in the auditory nerves of SF mice compared to the control group. (Figure 3C; red arrows). Quantitative analysis of myelin sheath thickness (see Method for specific procedures) indicated a significant reduction in myelin thickness in the SF mice compared to the control group (Figure 3D). These structural alterations in the ANF and their myelin sheaths may directly contribute to the observed functional deficits, specifically the decreased amplitude of ABR wave I and the prolonged latency observed in SF mice.

## 4 Discussion

Our research reveals a significant relationship between fragmented sleep and HHL, suggesting that fragmented sleep may cause subtle and underappreciated damage to the auditory system, highlighting the importance of maintaining healthy sleep habits to prevent auditory dysfunction. Our clinical analysis found that among individuals with normal audiograms, those with sleep disorders are more prone to speech recognition difficulties in noise. This association remained consistent even after adjusting for confounding factors and was observed across at least one level within the subgroups analyzed. Similar studies indicate that the China Health and Retirement Longitudinal Study found a significant association between insufficient nighttime sleep and an increased risk of self‐reported hearing loss (Cui et al. 2023). In contrast, data from the UK Biobank show no clear relationship between sleep duration and hearing risk, although this study relied on self‐reported hearing status rather than audiometric assessments (Yévenes‐Briones et al. 2023). This discrepancy may be attributed to differences in hearing assessment methods—self‐report versus audiograms. Our findings provide a more focused investigation into SiN perception, particularly among individuals with normal audiograms, allowing for a deeper exploration of the impact of sleep disorders.

Recent studies have made people aware that “I can hear, but not understand” may be caused not only by central lesions but also by lesions in the cochlear IHC‐ANF and the myelin of ANF (Kujawa and Liberman 2009; Wan and Corfas 2017). Due to the difficulty in conducting chronic SF experiments on humans and the difficulty in obtaining human temporal bone, we aim to build an SF model in mice to explore whether relevant lesions occur in the cochlea. Our experimental results confirm that 3 weeks of SF leads to lesions at the IHC‐ANF and in the myelin of ANF in mice. Although this finding cannot definitively indicate that individuals with normal audiograms but speech recognition difficulties in noise have similar pathological alterations, it at least provides a direction for explanation and offers a partial basis for providing neurotrophic treatment to these patients.

Traditionally, studies on the causes of HHL have focused primarily on noise exposure or aging (Monaghan et al. 2020; D et al. 2020; Liberman and Kujawa 2017; Gratias et al. 2021). Our previous study showed that 5 days of SF increased the sensitivity of ribbon synapses in the mouse cochlea to noise damage (Li et al. 2019). In this study, we found that extending the duration of SF led to a decrease in the number of ribbon synapses without any additional damaging effects. This suggests that the damage to the mouse auditory system may progress with the extension of SF time. The IHC‐ANF synapses are usually the first cochlear structures affected by aging or noise (Wu et al. 2019; Sergeyenko et al. 2013). If intervention cannot be performed in the early stage of SF, prolonged SF exposure may cumulatively induce progressive synaptic degeneration and demyelination of auditory nerve fibers, which could ultimately manifest as more profound or permanent forms of hearing impairment.

The deduction that patients with sleep disorders may have damage at the IHC‐ANF junction in the cochlea could also partially explain another auditory disease commonly associated with sleep disorders—tinnitus. Multiple studies have confirmed that sleep disorders are highly correlated with the occurrence and severity of tinnitus (Awad et al. 2024; Peng et al. 2023). This may be due to the reduced peripheral input following IHC‐ANF damage, resulting in compensatory gain enhancement in the subcortical and cortical centers of the ascending auditory pathway (Harris et al. 2022).

Myelin normally envelops nerve fibers, forming nodes of Ranvier that are vital for rapid nerve impulse conduction (Rasband and Peles 2021). The observed damage at myelin in our study could significantly slow down nerve conduction, thereby contributing to HHL. Clinical studies, including those examining peripheral neuropathies with demyelination such as Charcot‐Marie‐Tooth disease type 1, also reported an increased duration between ABR wave I and wave III (Rance et al. 2012). Actually, numerous studies have underscored the crucial role of sleep in the maintenance and renewal of nerve myelin (de Vivo and Bellesi 2019; Deantoni et al. 2023; Jamieson et al. 2020). Sleep fragmentation can lead to a thinning of the nerve myelin and an elongation of the internodal length of Ranvier's nodes, potentially slowing nerve impulse conduction and leading to cognitive deficits associated with sleep disturbances (Bellesi et al. 2018). Although previous studies have shown that 48‐hour sleep deprivation can lead to excessive autophagy and apoptosis of hippocampal neurons in mice (Cao et al. 2019), in this study, our count of spiral ganglion cells showed no significant difference between the SF group and the control group (Figure S3). This indicates that the spiral ganglion has not yet degenerated at this stage. This could be due to differences in modeling methods and durations.

Through what mechanisms might SF induce ribbon synapse damage and auditory nerve fiber demyelination? Research by Yadong Zheng et al. demonstrated that 7‐day chronic sleep restriction significantly elevates oxidative stress levels in murine brain tissue, with notable increases in lipid peroxidation products like 4‐HNE (Zheng et al. 2023). Alexandra Vaccaro et al. further observed that 2‐day and 5‐day sleep deprivation markedly enhanced intestinal reactive oxygen species (ROS) production (Vaccaro et al. 2020). These findings collectively suggest that SF may disrupt cochlear redox homeostasis, thereby damaging synapses and auditory nerve fibers. Additionally, neuroinflammation might be a plausible pathway linking SF to HHL. Di Sang et al. reported that 4‐day sleep deprivation triggers a cytokine storm‐like syndrome in mammals, resulting in fatal outcomes (Sang et al. 2023). In contrast, our study observed no such severe consequences—potentially attributable to the more subdued impact of sleep fragmentation and scheduled rest intervals in our protocol. Notably, G. Hurtado‐Alvarado et al. confirmed that sleep restriction induces chronic low‐grade systemic inflammation and compromises the blood‐brain barrier in C57BL/6 mice, elevating expression of neuroinflammatory markers (Iba‐1, A2A adenosine receptors, MMP‐9) in the hippocampus (Hurtado‐Alvarado et al. 2018). Analogously, the cochlea contains a vascular barrier termed the blood‐labyrinth barrier (BLB). SF may therefore instigate cochlear neuroinflammation via systemic inflammation‐mediated BLB disruption, ultimately impairing ribbon synapses and auditory nerve fibers. Elucidating these precise injury mechanisms holds significant positive implications for developing targeted interventions against sleep disorder‐induced auditory dysfunction.

Our research provides robust evidence of a link between SF and HHL, affirming the reliability and relevance of our experimental approach. These findings shed new light on how poor sleep affects the auditory system and suggest that the auditory nerve is more susceptible to damage from sleep disturbances than OHC. However, our findings cannot establish causality between SF and HHL. More robust exploration of this causal relationship will require subsequent molecular‐level mechanistic elucidation and interventional studies. In clinical research, as long as OHC function remains intact, standard pure‐tone audiometry is insufficiently sensitive to detect even diffuse neurodegeneration (Bourien et al. 2014; Ding et al. 1999; Lobarinas et al. 2013). This oversight underscores the potential for overlooking subtle hearing loss pathology in patients with sleep disorders. Future research should further investigate the specific mechanisms by which SF causes auditory dysfunction and explore therapeutic strategies to mitigate these effects through sleep recovery. This could have significant implications for preventing the harmful consequences of sleep fragmentation.

### 4.1 Limitation

The absence of electrophysiological validation (EEG/EMG) or actigraphy data represents a recognized limitation in the present study. Alexandra Vaccaro et al. applied a comparable sleep deprivation approach in their research, setting the rotational speed of the bar at 7 without scheduled pauses or rest intervals for mice. Their modeling paradigm demonstrated impacts on both REM and non‐REM sleep, with a particularly pronounced effect on REM (Vaccaro et al. 2020). This suggests that such methodologies may preferentially disrupt REM sleep. However, the precise effects on sleep architecture necessitate verification through EEG/EMG recordings in subsequent studies.

## Author Contributions

**Xiaodi Wang**: methodology, writing – review and editing, writing – original draft. **Bo Su**: methodology, writing – review and editing, writing – original draft. **Jiaxin Zhang**: methodology. **Yingqiang Li**: software, writing – original draft, conceptualization. **Liangqiang Zhou**: methodology. **Zhihui Du**: methodology. **Hanqi Chu**: supervision. **Huifang Liang**: supervision. **Dan Bing**: supervision, project administration, writing – review and editing.

## Statement of Significance

To address a critical knowledge gap regarding the relationship between sleep disorders and auditory anomalies, this study reveals a strong association between sleep disturbances and speech perception difficulties in noisy environments, even among individuals with normal hearing thresholds. Using a mouse model, we demonstrate that sleep fragmentation may lead to HHL, as indicated by cochlear synaptopathy and auditory nerve myelin damage. This may represent an underlying mechanism for speech perception difficulties related to sleep disturbances, in addition to central auditory system impairment. Moreover, we confirm that SF is another potential cause of HHL, apart from noise exposure and aging. This study is expected to both epidemiologically and mechanistically open new avenues for future research on the complex interplay between sleep and auditory function, and it provides evidence to guide the diagnosis and management of patients with sleep disorders who experience hearing difficulties.

## Disclosure

The authors have nothing to report.

## Conflicts of Interest

The authors declare no conflicts of interest.

## Peer Review

The peer review history for this article is available at https://publons.com/publon/10.1002/brb3.70778

## Supporting information




**Supplementary Material Figure‐S1**: brb370778‐sup‐0001‐FigureS1.png

**Supplementary Material Figure‐S2**: brb370778‐sup‐0002‐FigureS2.jpg

**Supplementary Material Figure‐S3**: brb370778‐sup‐0003‐FigureS3.jpg

**Supplementary Material**: brb370778‐sup‐0004‐SuppMat‐sleep‐fragmentation‐model.mp4

**Supplementary Material Table‐S1**: brb370778‐sup‐0005‐TableS1.docx

**Supplementary Material Table‐S2**: brb370778‐sup‐0006‐TableS2.docx

**Supplementary Material**: brb370778‐sup‐0007‐SuppMat.pdf

## Data Availability

The data underlying in the current study are available from the corresponding author upon reasonable request.
