# Supplementary figures and images for: Sleep Disturbances Associated With Hidden Hearing Loss: Insights From Human Data and a Mouse Model of Sleep Fragmentation
Source: Brain Behav. 2025 Aug 27;15(8):e70778. doi: 10.1002/brb3.70778 (PMC12381955; doi:10.1002/brb3.70778)

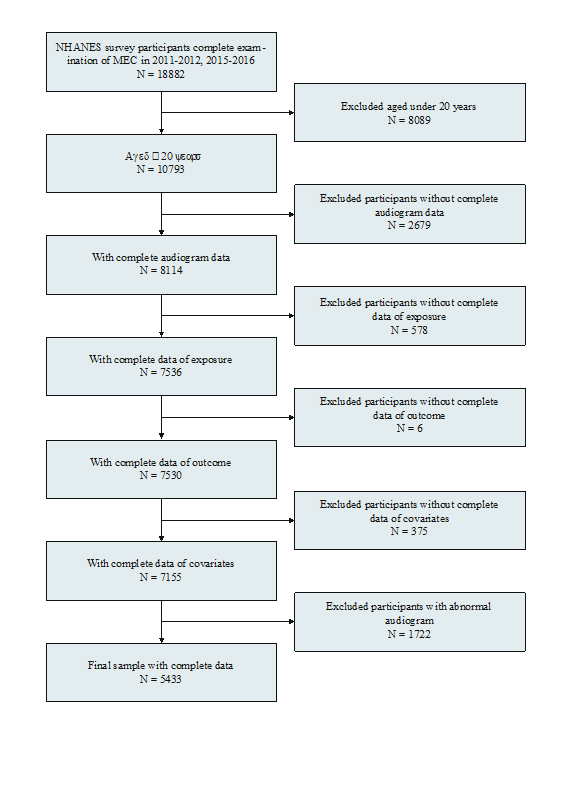

Supplement: Supplementary file 1 — Supplementary Material Figure‐S1: brb370778‐sup‐0001‐FigureS1.png [file BRB3-15-e70778-s001.png]

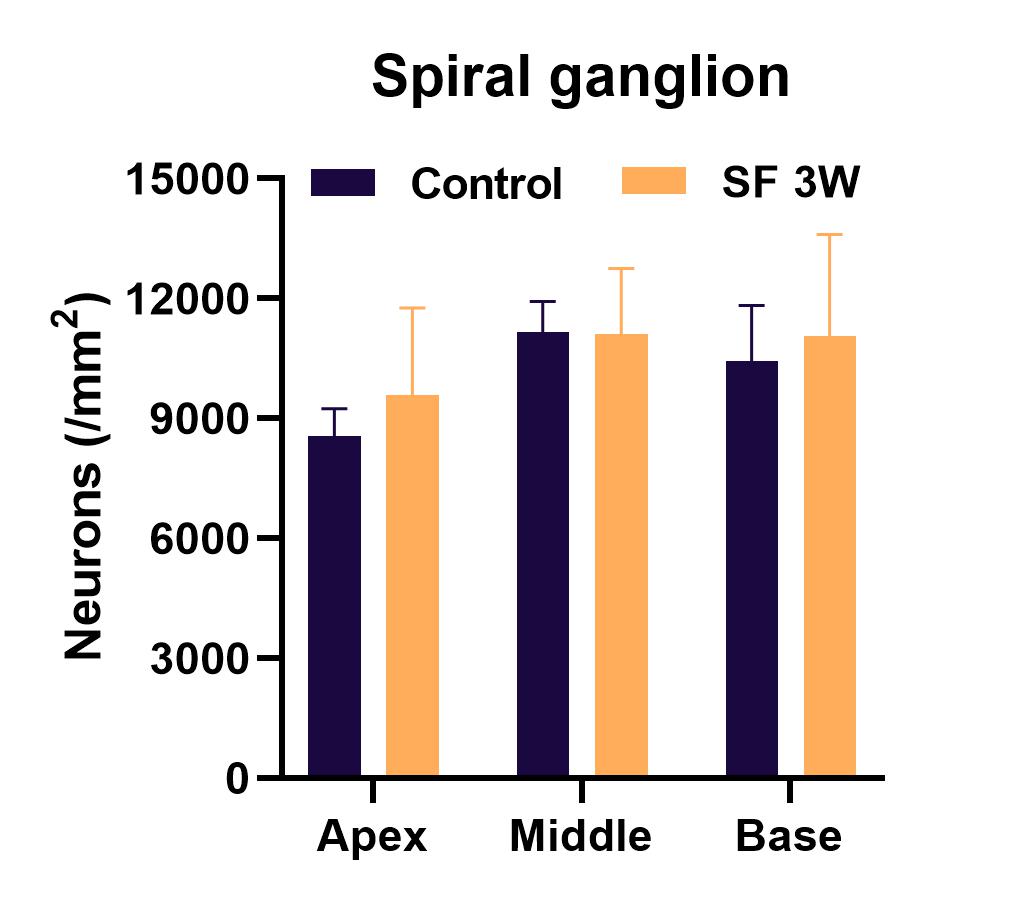

Supplement: Supplementary file 3 — Supplementary Material Figure‐S3: brb370778‐sup‐0003‐FigureS3.jpg [file BRB3-15-e70778-s002.jpg]
