# Supplementary material for: Sleep Disturbances Associated With Hidden Hearing Loss: Insights From Human Data and a Mouse Model of Sleep Fragmentation
Source: Brain Behav. 2025 Aug 27;15(8):e70778. doi: 10.1002/brb3.70778 (PMC12381955; doi:10.1002/brb3.70778)
Supplement: Supplementary file 5 — Supplementary Material Table‐S1: brb370778‐sup‐0005‐TableS1.docx [file BRB3-15-e70778-s004.docx]

**Table S1.** Characteristic of population included into the study

|  | **No. of participants (%)** | | |  |
| --- | --- | --- | --- | --- |
|  | **Total**  **(N = 5433)** | **Normal**  **(N = 4160)** | **SiN perception problem**  **(N = 1273)** | **P value** |
| **Age, y** |  |  |  |  |
| **< 60** | 4882(91.3) | 3751(91.4) | 1131(91.0) | 0.818 |
| **≥ 60** | 551(8.7) | 409(8.6) | 142(9.0) |  |
| **Gender** |  |  |  |  |
| **Male** | 2547(46.6) | 1964(46.1) | 583(48.2) | 0.351 |
| **Female** | 2886(53.4) | 2196(53.9) | 690(51.8) |  |
| **Race/ethnicity** |  |  |  |  |
| **Hispanic** | 1394(15.7) | 1081(16.2) | 313(14.1) | 0.001 |
| **Non-Hispanic white** | 1746(63.7) | 1287(63.1) | 459(65.8) |  |
| **Non-Hispanic black** | 1400(12.3) | 1067(12.3) | 333(12.3) |  |
| **Other** | 893(8.3) | 725(8.5) | 168(7.8) |  |
| **Educational level** |  |  |  |  |
| **Less than high school** | 940(11.8) | 681(11.3) | 259(13.6) | 0.001 |
| **High school** | 1132(18.9) | 817(17.6) | 315(23.3) |  |
| **More than high school** | 3361(69.2) | 2662(71.1) | 699(63.1) |  |
| **BMI** |  |  |  |  |
| **<25** | 1703(32.0) | 1323(32.3) | 380(30.8) | 0.644 |
| **≥25, <30** | 1669(32.1) | 1293(32.2) | 376(31.8) |  |
| **≥30** | 2061(35.9) | 1544(35.5) | 517(37.4) |  |
| **Hypertension** | 1481(23.8) | 1083(22.9) | 398(26.7) | 0.057 |
| **Cardiovascular disease history** | 221(3.0) | 141(2.4) | 80(5.1) | <0.001 |
| **Respiratory disease history** | 273(5.4) | 183(4.8) | 90(7.4) | 0.007 |
| **Diabetes mellitus** | 478(6.2) | 345(5.8) | 133(7.7) | 0.017 |
| **Noise exposure** | 1475(28.2) | 1038(25.7) | 437(36.4) | <0.001 |
| **Tinnitus** | 595(11.3) | 353(8.3) | 242(20.9) | <0.001 |
| **Sleep-related parameter** |  |  |  |  |
| **Sleep disturbance** | 756(13.2) | 475(10.8) | 281(20.9) | <0.001 |
| **Sleep trouble** | 1328(27.8) | 866(24.0) | 462(40.3) | <0.001 |

Abbreviation: SiN, speech in noise; BMI, body mass index.
