# Supplementary material for: Sleep Disturbances Associated With Hidden Hearing Loss: Insights From Human Data and a Mouse Model of Sleep Fragmentation
Source: Brain Behav. 2025 Aug 27;15(8):e70778. doi: 10.1002/brb3.70778 (PMC12381955; doi:10.1002/brb3.70778)
Supplement: Supplementary file 6 — Supplementary Material Table‐S2: brb370778‐sup‐0006‐TableS2.docx [file BRB3-15-e70778-s006.docx]

**Table S2.** Subgroup analysis of relationship between SiN perception problem and conditions of sleep

|  | Sleep disturbance | | Sleep trouble | |
| --- | --- | --- | --- | --- |
|  | OR (95% CI) | P for interaction | OR (95% CI) | P for interaction |
| Age |  |  |  |  |
| < 60 | 1.888(1.459-2.444) | 0.948 | 1.649(1.339-2.031) | 0.001 |
| ≥ 60 | 1.780(0.633- 5.000) |  | 8.601(3.495-21.166) |  |
| Gender |  |  |  |  |
| Male | 2.219(1.594-3.091) | 0.396 | 2.050(1.429-2.940) | 0.700 |
| Female | 1.669(1.198-2.325) |  | 1.831(1.363-2.458) |  |
| Race/ethnicity |  |  |  |  |
| Non white | 2.002(1.630-2.460) | 0.635 | 1.787(1.420-2.250) | 0.394 |
| White | 1.813(1.246-2.638) |  | 1.977(1.453-2.691) |  |
| Education |  |  |  |  |
| < 12 y | 1.029(0.608-1.742) | 0.023 | 2.466(1.191-5.105) | 0.681 |
| ≥ 12 y | 2.136(1.607-2.838) |  | 1.854(1.458-2.357) |  |
| BMI |  |  |  |  |
| <30 | 1.649(1.203-2.261) | 0.103 | 2.163(1.689-2.769) | 0.097 |
| ≥30 | 2.287(1.542-3.390) |  | 1.577(1.153-2.155) |  |

Abbreviation: BMI, body mass index.

Models were adjusted for age, sex, race/ethnicity, BMI, concurrent conditions (cardiovascular diseases, HBP, respiratory diseases, diabetes) and hearing related-status (noise exposure and tinnitus).
