# Supplementary material for: Sleep Disturbances Associated With Hidden Hearing Loss: Insights From Human Data and a Mouse Model of Sleep Fragmentation
Source: Brain Behav. 2025 Aug 27;15(8):e70778. doi: 10.1002/brb3.70778 (PMC12381955; doi:10.1002/brb3.70778)
Supplement: Supplementary file 7 — Supplementary Material: brb370778‐sup‐0007‐SuppMat.pdf [file BRB3-15-e70778-s003.pdf]

AUDIOMETRY – AUQ
Target Group: SPs 1+

AUQ.054 These next questions are about {your/SP's} hearing.

Which statement best describes {your/SP's} hearing (without a hearing aid or other listening devices)? Would you say {your/his/her} hearing is excellent, good, that {you have/s/he has} a little trouble, moderate trouble, a lot of trouble, or {are you/is s/he} deaf?

EXCELLENT	1
GOOD	2
A LITTLE TROUBLE	3
MODERATE HEARING TROUBLE	4
A LOT OF TROUBLE	5
DEAF	6
REFUSED	77
DON'T KNOW	99

HELP SCREEN:
Deaf means hearing loss so severe in both ears that hearing aids are insufficient to allow you to understand what people are saying.

Hearing Aid: A small electronic device that amplifies the sounds you hear. It is worn in or behind the ear to help you hear.

Other Listening Devices: Other listening devices are any device you use to help you hear. They are also called assistive listening devices. These are:

A pocket talker
An amplified telephone
An amplified or vibrating alarm clock
A light signaler for your doorbell
A TV headset
Closed-captioned TV
TTY (teletypewriter)
TDD (telecommunications device for the deaf)
A telephone relay service
A video relay service
A sign language interpreter

BOX 1A

CHECK ITEM AUQ.055:
IF SP AGE >= 20, AND SP AGE <= 69 AND AUQ.054 = 1, 7, 9, GO TO AUQ.100;
IF SP AGE >= 20, AND SP AGE <= 69 AND AUQ.054 = 2, 3, 4, 5 OR 6, CONTINUE.
IF SP AGE > 69, GO TO AUQ.136.
OTHERWISE, END OF SECTION.

AUQ.060 These next questions refer to hearing without the use of a hearing aid or any other listening devices. If {you have/SP has} one ear that is better than the other, please answer the questions for the hearing in {your/SP's} better ear.

Can {you/SP} usually **hear and understand** what a person says without seeing his or her face if that person **whispers** to {you/him/her} from across a quiet room?

YES	1	(AUQ.100)
NO	2	
REFUSED	7	
DON'T KNOW	9	

AUQ.070 Can {you/SP} usually **hear and understand** what a person says without seeing his or her face if that person **talks in a normal voice** to {you/him/her} from across a quiet room?

YES	1	(AUQ.100)
NO	2	
REFUSED	7	
DON'T KNOW	9	

AUQ.080 Can {you/SP} usually **hear and understand** what a person says without seeing his or her face if that person **shouts** to {you/him/her} from across a quiet room?

YES	1	(AUQ.100)
NO	2	
REFUSED	7	
DON'T KNOW	9	

AUQ.090 Can {you/SP} usually **hear and understand** what a person says without seeing his or her face if that person **speaks loudly** into {your/his/her} **better ear**?

INTERVIEWER: IF THE INTERVIEWEE HEARS BETTER IN ONE EAR THAN THE OTHER, RECORD THE RESPONSE FOR SPEAKING LOUDLY INTO THE BETTER EAR.

YES	1
NO	2
REFUSED	7
DON'T KNOW	9

AUQ.100 How often {do you/does SP} find it difficult to follow a conversation if there is background noise, for example, when other people are talking, TV or radio is on, or children are playing? Would you say...

HAND CARD AUQ1

Always,	1
Usually,	2
About half the time,	3
Seldom, or	4
Never?	5
REFUSED	7
DON'T KNOW	9

AUQ.110 How often does {your/SP's} hearing cause {you/him/her} to feel frustrated when talking to members of {your/his/her} family or to friends? Would you say...

HAND CARD AUQ1

Always,	1
Usually,	2
About half the time,	3
Seldom, or	4
Never?	5
REFUSED	7
DON'T KNOW	9

AUQ.136 {Have you/Has SP} **ever** had 3 or more ear infections? Please include ear infections {you/he/she} may have had when {you were/he was/she was} a child.

YES	1
NO	2
REFUSED	7
DON'T KNOW	9

AUQ.138 {Have you/Has SP} **ever** had a tube placed in {your/his/her} ear to drain the fluid from {your/his/her} ear?

YES	1
NO	2
REFUSED	7
DON'T KNOW	9

BOX 3

CHECK ITEM AUQ.137:
IF SP AGE > 69, GO TO AUQ.146.
OTHERWISE, CONTINUE.

AUQ.144 A hearing test by a specialist is one that is done in a sound proof booth or room, or with headphones. Hearing specialists include audiologists, ear nose and throat doctors, and trained technicians or occupational nurses. When was the last time {you had/SP had} {your/his/her} hearing tested by a hearing specialist?

READ CATEGORIES IF NECESSARY

LESS THAN A YEAR AGO	1	
1 YEAR TO 4 YEARS AGO	2	
5 TO 9 YEARS AGO	3	
TEN OR MORE YEARS AGO	4	
NEVER	5	
REFUSED	7	
DON'T KNOW	9	

AUQ.146 {Have you/Has SP} **ever** worn a hearing aid or cochlear implant?

YES	1	
NO	2	(BOX 4)
REFUSED	7	(BOX 4)
DON'T KNOW	9	(BOX 4)

HELP SCREEN:
Hearing Aid: A small electronic device that amplifies the sounds you hear. It is worn in or behind the ear to help you hear.

Cochlear Implant: A cochlear implant is an electrical device that a surgeon puts in your ear. It helps you hear by sending sounds directly to the brain. It is used only when you are almost totally deaf.

AUQ.148 Which was it?

CODE ALL THAT APPLY

CAPI INSTRUCTION:
IF BOTH RESPONSE OPTIONS 1 AND 2 ARE SELECTED, GO TO AUQ.152.

A HEARING AID	1	
A COCHLEAR IMPLANT	2	(BOX 4)
REFUSED	7	(BOX 4)
DON'T KNOW	9	(BOX 4)

AUQ.152 In the **past 12 months**, how often {have you/has SP} worn a hearing aid?

HAND CARD AUQ1

ALWAYS	1
USUALLY	2
ABOUT HALF THE TIME	3
SELDOM	4
NEVER	5
REFUSED	7
DON'T KNOW	9

HELP SCREEN:
Hearing Aid: A small electronic device that amplifies the sounds you hear. It is worn in or behind the ear to help you hear.

BOX 4

CHECK ITEM AUQ.390:
IF SP AGE > 69, GO TO END OF SECTION.
OTHERWISE, CONTINUE.

AUQ.154 {Have you/Has SP} **ever** used assistive listening devices (ALDs), such as FM systems, closed-captioned television, amplified telephone, relay services, or a sign-language interpreter?

YES	1	
NO	2	
REFUSED	7	
DON'T KNOW	9	

HELP SCREEN:
Assistive Listening Devices: These are any device you use to help you hear. Other examples include:

TTY (teletypewriter)
TDD (telecommunications device for the deaf)
A pocket talker
An amplified or vibrating alarm clock
A light signaler for your doorbell
A TV headset

AUQ.191 In the **past 12 months**, {have you/has SP} been bothered by ringing, roaring, or buzzing in {your/his/her} ears or head **that lasts for 5 minutes or more**?

YES	1	
NO	2	(AUQ.300)
REFUSED	7	(AUQ.300)
DON'T KNOW	9	(AUQ.300)

HELP SCREEN:
Tinnitus (tin-uh-tus) is the medical term for ringing, roaring or buzzing in the ears or head.

AUQ.250 How long {have you/has SP} been bothered by this ringing, roaring, or buzzing in {your/his/her} ears or head?

READ CATEGORIES IF NECESSARY

LESS THAN THREE MONTHS	1
THREE MONTHS TO A YEAR	2
1 TO 4 YEARS	3
5 TO 9 YEARS	4
TEN OR MORE YEARS	5
REFUSED	7
DON’T KNOW	9

AUQ.255 **In the past 12 months**, how often {have you/has SP} had this ringing, roaring, or buzzing in {your/his/her} ears or head? Would you say...

almost always,	1
at least once a day,	2
at least once a week,	3
at least once a month, or	4
less frequently than once a month?	5
REFUSED	7
DON'T KNOW	9

AUQ.260 {Are you/Is SP} **bothered** by ringing, roaring, or buzzing in {your/his/her} ears or head **only** after listening to loud sounds or loud music?

YES	1
NO	2
REFUSED	7
DON'T KNOW	9

AUQ.270 {Are you/Is SP} **bothered** by ringing, roaring, or buzzing in {your/his/her} ears or head when going to sleep?

YES	1
NO	2
REFUSED	7
DON'T KNOW	9

AUQ.280 How much of a problem is this ringing, roaring, or buzzing in {your/his/her} ears or head? Would you say...

No problem,	1
A small problem,	2
A moderate problem,	3
A big problem, or	4
A very big problem?	5
REFUSED	7
DON'T KNOW	9

AUQ.300 This next question is about {your/SP's} use of firearms that {you/he/she} may have used for target shooting, hunting, for {your/his/her} job or in military service. {Have you/Has SP} ever used firearms for any reason?

YES	1	
NO	2	(AUQ.331)
REFUSED	7	(AUQ.331)
DON'T KNOW	9	(AUQ.331)

HELP SCREEN:
Firearms include pistols, shotguns, rifles, and other types of guns. Do not include BB or pellet guns.

AUQ.310 How many **total** rounds {have you/has SP} ever fired?

READ CATEGORIES IF NECESSARY

INTERVIEWER: ONE ROUND EQUALS ONE SHOT. INCLUDE TARGET SHOOTING, HUNTING, YOUR JOB AND MILITARY SERVICE.

1 TO LESS THAN 100 ROUNDS	1
100 TO LESS THAN 1000 ROUNDS	2
1000 TO LESS THAN 10,000 ROUNDS	3
10,000 TO LESS THAN 50,000 ROUNDS	4
50,000 ROUNDS OR MORE	5
REFUSED	7
DON'T KNOW	9

AUQ.320 How often {did you/did SP} wear hearing protection devices (ear plugs, ear muffs) when shooting firearms?

INTERVIEWER: PROTECTIVE HEARING DEVICES INCLUDE PLUGS AND EARMUFFS.

HAND CARD AUQ1

ALWAYS	1
USUALLY	2
ABOUT HALF THE TIME	3
SELDOM	4
NEVER	5
REFUSED	7
DON'T KNOW	9

AUQ.331 These next questions are about noise exposure at work. First we are going to ask about **loud** noise. **Loud** means so loud that {you/s/he} must speak in a raised voice to be heard by someone three feet away when not using hearing protection. After that we will ask about **very loud noise**. **Very loud noise** is noise that is so loud {you have/he has/she has} to shout in order to be understood by someone standing 3 feet away from {you/him/her} when not using hearing protection.

{Have you/Has SP} **ever had a job, or combination of jobs where** {you were/s/he was} exposed to loud sounds or noise for **4 or more hours a day, several days a week**? (Loud means so loud that {you/s/he} must speak in a raised voice to be heard.)

YES	1	
NO	2	(AUQ.370)
NEVER WORKED	3	(AUQ.370)
REFUSED	7	(AUQ.370)
DON'T KNOW	9	(AUQ.370)

AUQ.340 For how many months or years {have you/has SP} been exposed at work to loud sounds or noise for 4 or more hours a day, several days a week?

READ CATEGORIES IF NECESSARY

LESS THAN 3 MONTHS	1
3 TO 11 MONTHS	2
1 TO 2 YEARS	3
3 TO 4 YEARS	4
5 TO 9 YEARS	5
10 TO 14 YEARS	6
15 OR MORE YEARS	7
REFUSED	77
DON'T KNOW	99

AUQ.350 In {your/SP's} work {were you/was he/was she} exposed to **very loud noise**? (**Very loud noise** is noise that is so loud {you have/he has/she has} to shout in order to be understood by someone standing 3 feet away from {you/him/her}.)

YES	1	
NO	2	(AUQ.370)
REFUSED	7	(AUQ.370)
DON'T KNOW	9	(AUQ.370)

AUQ.361 Please give me the total number of months or years for all jobs where this has happened.

READ CATEGORIES IF NECESSARY

LESS THAN 3 MONTHS	1
3 TO 11 MONTHS	2
1 TO 2 YEARS	3
3 TO 4 YEARS	4
5 TO 9 YEARS	5
10 TO 14 YEARS	6
15 OR MORE YEARS	7
NOT EXPOSED	8
REFUSED	77
DON'T KNOW	99

AUQ.370 **Outside of a job,** {have you/has SP} **ever** been exposed to **very loud noise or music** for **10 or more hours a week**? This is noise so loud that {you have/s/he has} to shout to be **understood or heard 3 feet away**. Examples are noise from power tools, lawn mowers, farm machinery, cars, trucks, motorcycles, motor boats or loud music.

YES	1
NO	2
REFUSED	7
DON'T KNOW	9

AUQ.381 **In the past 12 months, how often** {did you/did SP} wear hearing protection devices (ear plugs, ear muffs) when exposed to very loud sounds or noise outside of work? {Do not include the noise from firearms we already talked about.}

INTERVIEWER: PROTECTIVE HEARING DEVICES INCLUDE PLUGS AND MUFFS.

HAND CARD AUQ2

ALWAYS	1
USUALLY	2
ABOUT HALF THE TIME	3
SELDOM	4
NEVER	5
NO NOISE EXPOSURE PAST 12 MONTHS	6
REFUSED	77
DON'T KNOW	99

CAPI INSTRUCTION:
IF AUQ.300 = 1/YES, DISPLAY 'Do not include the noise from firearms we already talked about.'
